# Association between Dental Scaling and Reduced Risk of End-Stage Renal Disease: A Nationwide Matched Cohort Study

**DOI:** 10.3390/ijerph18178910

**Published:** 2021-08-24

**Authors:** Yu-Hsiang Chung, Hsien-Cheng Kuo, Hsin-Yi Liu, Mei-Yi Wu, Wei-Jen Chang, Jui-Tai Chen, Yih-Giun Cherng, Tzeng-Ji Chen, Ying-Xiu Dai, Hsiang-Ling Wu, Wan-Chi Liu, Ying-Hsuan Tai

**Affiliations:** 1 Department of Anesthesiology, Shuang Ho Hospital, Taipei Medical University, New Taipei City 23561, Taiwan; 20270@s.tmu.edu.tw (Y.-H.C.); 19585@s.tmu.edu.tw (H.-C.K.); 18384@s.tmu.edu.tw (H.-Y.L.); 19240@s.tmu.edu.tw (J.-T.C.); stainless@s.tmu.edu.tw (Y.-G.C.); 2 Department of Anesthesiology, School of Medicine, College of Medicine, Taipei Medical University, Taipei 11031, Taiwan; 3 Division of Nephrology, Department of Internal Medicine, Shuang Ho Hospital, Taipei Medical University, New Taipei City 23561, Taiwan; 09643@s.tmu.edu.tw; 4 Division of Nephrology, Department of Internal Medicine, Taipei Medical University, Taipei 11031, Taiwan; 5 Department of Dentistry, Shuang Ho Hospital, Taipei Medical University, New Taipei City 23561, Taiwan; cweijen1@tmu.edu.tw; 6 School of Dentistry, College of Oral Medicine, Taipei Medical University, Taipei 11031, Taiwan; 7 Department of Family Medicine, Taipei Veterans General Hospital, Taipei 11217, Taiwan; tjchen@vghtpe.gov.tw; 8 School of Medicine, National Yang Ming Chiao Tung University, Taipei 11221, Taiwan; daiinxiu@gmail.com (Y.-X.D.); hlwu9@vghtpe.gov.tw (H.-L.W.); 9 Department of Dermatology, Taipei Veterans General Hospital, Taipei 11217, Taiwan; 10 Department of Anesthesiology, Taipei Veterans General Hospital, Taipei 11217, Taiwan

**Keywords:** cardiovascular event, dental prophylaxis, dialysis, renal failure

## Abstract

Periodontitis is prevalent in patients with chronic kidney disease (CKD) and is also associated with kidney function decline. It is unclear whether dental scaling treatment prevents the progression of CKD. In a nationwide cohort study, Taiwan’s National Health Insurance Research Database was used to select people with CKD. Propensity score-matching procedures were performed to compare the long-term risk of end-stage renal disease (ESRD) between CKD patients with and without the receipt of dental scaling. A total of 33,637 matched pairs with CKD were included, with 503,373 person-years of follow-up for analyses. Dental scaling was significantly associated with a lower risk of ESRD (adjusted hazard ratio (aHR): 0.83, 95% confidence interval (CI): 0.77–0.90). In addition, there was a dose-dependent relationship between the frequency of dental scaling and a reduced risk of ESRD. Dental scaling was also linked to reduced risks of major adverse cardiovascular events (aHR: 0.91, 95% CI: 0.87–0.95), sepsis (aHR: 0.81, 95% CI: 0.77–0.85), and all-cause mortality (aHR: 0.81, 95% CI: 0.76–0.87). Dental scaling was significantly associated with lower risks of progression to ESRD in patients with CKD. Regular dental scaling may serve as a prophylactic measure for kidney function decline.

## 1. Introduction

Chronic kidney disease (CKD) is a leading cause of mortality and morbidity worldwide, with a staggering prevalence of nearly 700 million cases in 2018 [1]. CKD leads to progressive and irreversible nephron loss, end-stage renal disease (ESRD) and premature mortality [2]. Prevention of kidney function decline and its relevant complications are cornerstones in the management of CKD [3]. However, there are currently few effective therapies for preventing the progression of CKD [4].

Periodontal disease is noticeably prevalent and severe in CKD sufferers [5]. Mounting evidence revealed that chronic periodontitis may adversely impact kidney function [6,7], increase the risk of cardiovascular disease [8,9], and shorten overall survival in CKD patients [6,10,11]. In addition, the severity of periodontal diseases is significantly correlated with the severity of CKD [12,13,14,15]. The transient bacteremia and inflammatory status induced by periodontitis are regarded as the culprit for the deterioration of kidney function and the development of systemic comorbidities [12,16,17,18,19]. Gingivitis facilitates the entry of oral microorganisms into the circulation and thereafter induces a systemic inflammatory response [12]. Studies have shown that systemic inflammation may contribute to kidney function loss through the mechanisms of elevated urinary albumin excretion, glomerular hypo- and hyper-filtration, endothelial dysfunction, anemia, and erythropoietin resistance [17,20]. In addition, periodontal inflammation increases the production of reactive oxygen species, which is a key contributor to the pathogenesis of CKD [21].

Non-surgical periodontal therapy (e.g., dental scaling) has been demonstrated to reduce the serum levels of inflammatory markers [22,23], and improve periodontal status [22,23,24] and endothelial function [23]. Furthermore, periodontal care and treatment were linked to longer survival [25] and an improved glomerular filtration rate [26,27] in patients with chronic periodontitis. However, it remains uncertain whether periodontal therapy is beneficial in preventing kidney function decline and its related complications [22,23,24,25,26,27]. The protective effect of dental scaling on CKD patients is not fully clarified due to the study limitations of previous reports, including small numbers of subjects [22,23,24,25,26,27], inadequate adjustment for confounding factors [26,27], an incomprehensive evaluation of CKD outcomes [22,23,24,25,26,27], and the study being restricted to patients with ESRD [22,24,25,27] or from single institutions [22,23,24,26,27]. Overall, current evidence is insufficient and inconclusive to confirm or refute the therapeutic role of dental scaling in the prevention of CKD progression.

This study aimed to investigate the effect of dental scaling, a common form of periodontal care, on the long-term risk of ESRD and major complications in CKD patients. Specifically, we hypothesized that the receipt of dental scaling was associated with lower risks of progression to ESRD, major adverse cardiovascular events (MACE), infections, and all-cause mortality in CKD patients.

## 2. Materials and Methods

### 2.1. Source of Data

We used Taiwan’s National Health Insurance Research Database (NHIRD) to conduct a nationwide, population-based cohort study. The National Health Insurance program was launched in March 1995 and covered more than 99% of the 23.5 million residents in Taiwan. The NHIRD includes comprehensive information about the insured people, including demographic features (date of birth, sex, and residential location) and medical claims data (outpatient and inpatient care, physicians’ diagnoses, medical prescriptions, procedures, and expenditures). Research articles based on the NHIRD and the related validation have been widely accepted in prominent scientific journals worldwide [28,29,30,31]. This study used three Longitudinal Health Insurance Databases (LHID2000, LHID2005, and LHID2010), which randomly sampled 1 million subjects from the original NHIRD in the years 2000, 2005, and 2010, respectively. The representativeness of LHIDs has been validated by Taiwan’s National Health Research Institutes [32].

### 2.2. Patient Selection

Inclusion criteria were patients aged ≥20 years who had a record of CKD held in the LHID2000, LHID2005, and LHID2010 databases between 1 January 2000 and 30 December 2011. We used the International Classification of Diseases, 9th Revision, Clinical Modification (ICD-9-CM) codes to identify CKD cases (Supplementary Table S1). Only patients with a CKD diagnosis that was made at least 2 times were included, in order to better ascertain the CKD patients [33]. Exclusion criteria were people receiving renal replacement therapy before the index date. We defined the date of the first diagnosis of CKD as the index date. In Taiwan’s Health Insurance reimbursement regulations, beneficiaries were offered free dental scaling treatment (administration code: 91003, 91004, 91005, 91017, 91103, 91104) every 6 months. Patients with CKD were further divided into those with or without dental scaling treatment within 24 months before the index date. We matched each patient receiving dental scaling to an individual without a record of dental scaling, using a greedy propensity score matching algorithm within a tolerance limit of 0.05 and without replacement, to balance the distributions of age, sex, insurance premium, coexisting diseases, and lifestyle factors between groups [34].

### 2.3. Covariates for Adjustment

Insurance premiums were categorized into USD 0–500, USD 501–800, and >USD 800 per month. Patients with concurrent usage of erythropoiesis-stimulating agents covered by health insurance were determined as cases with stage 5 CKD. In the reimbursement regulations of Taiwan’s National Health Insurance, erythropoiesis-stimulating agents can be initiated when non-dialyzed CKD patients have a serum creatinine level >530 μmol/L (approximately equivalent to stage 5 CKD) [35]. The ICD-9-CM codes of physicians’ diagnoses within 24 months before the index date were employed to identify a history of the following comorbidities: hypertension, diabetes mellitus, ischemic heart disease, atherosclerosis, cardiac dysrhythmias, heart failure, liver cirrhosis, chronic obstructive pulmonary disease, cerebrovascular disease, dyslipidemia, malignancies, and mental disorders (Supplementary Table S1). Lifestyle factors included smoking disorders, alcohol abuse, malnutrition, and obesity. These covariates were selected, based on data availability, physiological plausibility, and the existing literature.

Our analysis also considered those dental procedures performed within 24 months before the index date, including subgingival curettage, periodontal flap surgery, tooth extraction, odontectomy, and emergent dental care. We included the subjects’ current medications prescribed within 24 months before the index date, including systemic antibiotics (amoxicillin, cephalexin, clindamycin, and metronidazole) [36], statins (atorvastatin, fluvastatin, lovastatin, pitavastatin, pravastatin, rosuvastatin, and simvastatin) [31], metformin [31], and influenza vaccinations [31].

### 2.4. Primary and Secondary Outcomes

The primary outcome is considered to be the progression to ESRD, defined as the date patients began dialysis for at least 90 days. In Taiwan’s Health Insurance reimbursement regulations, patients can undergo long-term dialysis only when their estimated glomerular filtration rate drops below 5 mL/min/1.73 m^2^. Secondary outcomes are all-cause mortality and hospitalizations for MACE, septicemia or sepsis, urinary tract infection, pyelonephritis, and acute renal failure (Supplementary Table S1). MACE include acute myocardial infarction, new-onset heart failure, stroke, and cardiac dysrhythmias. The included subjects were followed up until 31 December 2013.

### 2.5. Statistical Analysis

A non-parsimonious multivariable logistic regression model was used to estimate a propensity score for subjects with or without dental scaling treatment. The distributions of the baseline characteristics in the matched sample were compared using the standardized difference [37]. The cumulative incidence of ESRD was illustrated with Kaplan–Meier curves and compared between groups using log-rank tests. The adjusted hazard ratio (aHR) and 95% confidence interval (CI) of selected outcomes were calculated by multivariable Cox proportional hazard regression models. All the collected covariates were incorporated into multivariable models to minimize potential confounding effects, including age, sex, insurance premium, lifestyle factors, coexisting diseases, concurrent medications, and dental procedures (Table 1). We also conducted stratified analyses by age, sex, comorbid periodontal disease, hypertension, and diabetes, uses of systemic antibiotics, statins, and metformin, and different dental procedures. The interaction between the protective impact of dental scaling treatment and covariates was examined for the risk of ESRD, including age, sex, comorbid periodontal disease, hypertension, and diabetes, the usage of systemic antibiotics, statins, and metformin. Finally, as regards sensitivity tests, stepwise backward variable elimination procedures were applied with an entry probability of 0.05 and removal probability of 0.1, to determine the independent factors associated with progression to ESRD. A two-sided level of 0.05 was considered statistically significant. All the statistical analyses were conducted using the statistical analysis system (SAS), version 9.4 (SAS Institute Inc., Cary, NC, USA).

## 3. Results

### 3.1. Baseline Patient Characteristics

**Figure 1:**
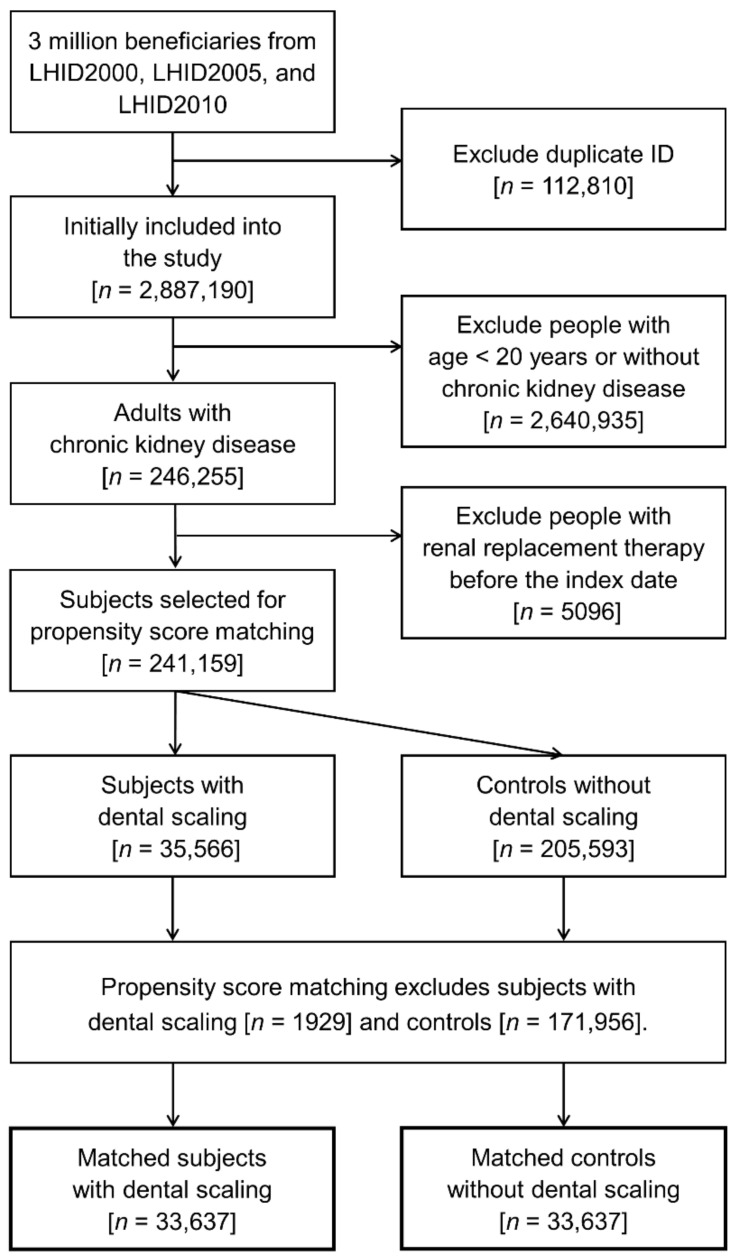
Flow diagram for patient selection.

The matching procedure generated 33,637 matched pairs (Figure 1). The follow-up interval was a median of 8.1 years (interquartile range 5.0–10.1), and the cumulative time at risk of all matched subjects was 503,373 person-years. Table 1 shows the baseline characteristics of the included subjects with and without dental scaling treatment. The distributions of demographics, coexisting diseases, and lifestyle factors were well balanced after matching. In the dental scaling group, 24,306 (72.3%), 7003 (20.8%), and 2328 (6.9%) subjects received dental scaling once, twice, and ≥three times within 24 months before the index date.

### 3.2. Dental Scaling and Progression to ESRD

**Figure 2:**
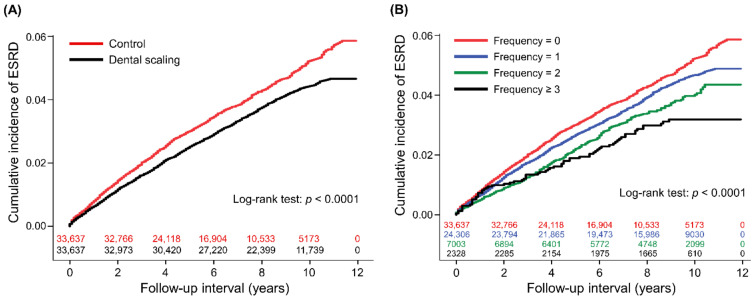
Cumulative incidences of ESRD between people with and without dental scaling (**A**), and with a varying frequency of dental scaling (**B**), with the number of subjects at risk.

The 1-year, 3-year, and 5-year cumulative incidences of ESRD were 0.6% (95% CI: 0.6–0.6), 1.6% (1.4–1.8), and 2.5% (2.3–2.7) for people with dental scaling, and 0.8% (0.8–0.8), 2.0% (1.8–2.2), and 3.0% (2.8–3.2) for controls (Figure 2A). The duration between index date and ESRD was median 3.8 years (interquartile range 1.7–6.3) for people with dental scaling and 2.8 years (1.2–5.0) for controls. After controlling for covariates, dental scaling was significantly associated with a lower risk of ESRD (aHR: 0.83) (Table 2). A backward variable elimination analysis showed similar results (Table 3). There was a graded relationship between the frequency of dental scaling and a reduced risk of ESRD, 1 vs. 0 (aHR: 0.85, 95% CI: 0.78–0.93, *p* = 0.0004), 2 vs. 0 (aHR: 0.80, 95% CI: 0.70–0.92, *p* = 0.0017), and ≥3 vs. 0 (aHR: 0.67, 95% CI: 0.52–0.86, *p* = 0.0017) (Figure 2B). Subgroup analysis showed that the lower risk of ESRD associated with dental scaling was significant in those who were aged <65 years, had no periodontal disease, and had no recorded use of systemic antibiotics (Table 4). Supplementary Table S2 shows the risk of ESRD in patients who received dental scaling, who also had various dental treatments. There was a significant interaction between the protective effect of dental scaling and some covariates, including age, hypertension, diabetes mellitus, the use of statins and metformin (Supplementary Table S3). The relationship between dental scaling and a reduced risk of ESRD was virtually unchanged after considering age, hypertension, diabetes mellitus, the use of statins and metformin as interacting factors with dental scaling treatment (*p* <0.0001 after the adjustment for hazard ratio interaction).

### 3.3. Dental Scaling and MACE

The 1-year, 3-year, and 5-year cumulative incidences of MACE were 3.2% (95% CI: 3.0–3.4), 7.0% (6.8–7.2), and 10.4% (10.0–10.8) for people with dental scaling, and 3.6% (3.4–3.8), 7.6% (7.4–7.8), and 11.1% (10.7–11.5) for controls. The duration between index date and MACE was a median of 3.6 years (interquartile range 1.4–6.3) for people with dental scaling and 2.6 years (0.9–5.1) for controls. After adjusting for covariates, dental scaling treatment was significantly associated with a lower risk of MACE (aHR: 0.91) (Table 2).

### 3.4. Dental Scaling and All-Cause Mortality

The 1-year, 3-year, and 5-year cumulative incidences of all-cause mortality were 0.5% (95% CI: 0.5–0.5), 1.4% (1.2–1.6), and 2.4% (2.2–2.6) for people with dental scaling, and 0.8% (0.8–0.8), 2.0% (1.8–2.2), and 3.4% (3.2–3.6) for controls. The duration between the index date and all-cause mortality was a median of 5.6 years (interquartile range 2.9–8.0) for people with dental scaling and 3.8 years (1.8–6.4) for controls. After adjusting for covariates, dental scaling was significantly associated with a lower risk of all-cause mortality (aHR: 0.81) (Table 2).

### 3.5. Dental Scaling and Infections

Dental scaling was significantly associated with reduced risks of hospitalization for septicemia or sepsis (aHR: 0.81), urinary tract infection (aHR: 0.87), pyelonephritis (aHR: 0.87), and acute renal failure (aHR: 0.76) (Table 2).

## 4. Discussion

Our study showed that dental scaling was significantly associated with lower risks of progression to ESRD, MACE, infections, and all-cause mortality in patients with CKD. In addition, there existed a dose-dependent relationship between the frequency of dental scaling and a reduced risk of ESRD. This study had several strengths in investigating the putative effect of dental scaling on CKD outcomes. First, our analyses were based on a nationwide database, which covered a wide variety of individuals with divergent demographic and clinical characteristics. This increases the generalizability of the study results. Second, the longitudinal study design clarified the temporal relationship between dental scaling and CKD outcomes. Third, we used propensity score matching to minimize the potential confounding effects of demographic features, comorbidities, and lifestyle factors. Our results indicated that dental scaling may protect CKD patients against major morbidity and mortality, providing an important prophylactic strategy to improve the prognosis of CKD.

To our knowledge, this is the first large-scale observational study to comprehensively evaluate the impact of dental scaling on the medical outcomes of CKD, instead of laboratory markers [22,23,24] or kidney function alone [26,27]. Previous studies struggled with a small sample size [22,23,24,25,26,27], their exploratory nature [24,26], and being restricted to patients with ESRD [22,24,25,27]. Two meta-analyses assessed inflammatory markers [38] and kidney function [39], while lacking adequate high-quality evidence from clinical trials. In a multinational cohort study, Palmer and colleagues reported that preventive oral hygiene practices could decrease all-cause mortality but not cardiovascular mortality in hemodialysis patients [25]. However, this study did not evaluate MACE or infections, and it is difficult to apply these results to non-dialyzed CKD patients.

Several mechanisms may contribute to the protective effects of dental scaling for CKD patients. Systemic inflammatory status induced by periodontitis was correlated with the deterioration of kidney function and the development of cardiovascular disease [12,16,17,18,19]. Non-surgical periodontal treatment is vital in the treatment of periodontal disease [40] and may effectively ameliorate systemic inflammation [22,23,24]. Moreover, the receipt of dental scaling treatment may reflect the better socioeconomic status and medical knowledge, attitudes, and practice in CKD patients, which may underlie the reduced complications associated with dental scaling [41,42]. Our subgroup analysis demonstrated that the reduced risk of ESRD associated with dental scaling was significant in those aged <65 years, with no periodontal disease, no hypertension, and no recorded use of systemic antibiotics. We raised the following pathophysiological mechanisms for these findings. First, geriatric patients were more likely to have multiple and more severe comorbidities and have reduced kidney function, which might override the beneficial effects of dental scaling [43]. Besides, the elderly patients had a lower number of existing permanent teeth and required dentures, which might be associated with a lower need for dental scaling [44]. Second, the protective effect of dental scaling against ESRD was only observed in patients without a periodontist, which indicated that dental scaling might serve as a preventive rather than a therapeutic measure for CKD patients. A clinical trial showed that full-mouth scaling may significantly stimulate the release of circulating inflammatory mediators for patients with moderate to severe chronic periodontitis [45]. It remains unclear whether the acute inflammatory response induced by dental scaling affects kidney function in periodontitis sufferers. Third, hypertension is an established risk factor for kidney function decline [46]. Hypertensive kidney injury may conceal the protective effect of dental scaling against ESRD when under long-term observation. Fourth, systemic antibiotics could act as a pharmacological treatment for periodontitis [47]. Therefore, prior usage of antibiotics may attenuate the kidney injury from periodontitis and overshadow the protective impact of dental scaling.

More efforts are still needed to clarify the associative or causal relationship between dental scaling and CKD outcome, due to the observational nature of our study. Our findings suggested that promoting regular dental scaling may serve as a potential measure to reduce the complication of CKD, in addition to the conventional practices of medication, diet control, and lifestyle modification [48]. It is reasonable to routinely refer patients with CKD for dental and periodontal examination, oral hygiene education, and a dental scaling service. An interdisciplinary approach involving nephrologists and dentists may effectively prevent kidney function decline and improve the clinical outcome in this vulnerable population. Our results implied that patients receiving dental scaling ≥3 times within 2 years had the lowest risk of ESRD compared to other subgroups. It is important to elucidate the optimal frequency of routine scaling provided for CKD patients to maintain periodontal health. However, there is still no consensus on this issue to date [22,23,24,25,26,27]. Future studies should focus on the favorable content and frequency of periodontal care for CKD patients, and separately investigate those with and without periodontitis and varying disease severity.

There are several limitations to our study. First, our data did not contain information about physical measures (e.g., periodontal parameters and tooth loss), biochemical laboratory tests (e.g., baseline glomerular filtration rates and the level of inflammatory markers), socioeconomic factors, and antihypertensive medications. Second, our study did not consider the pathophysiology of CKD and causes of death, due to data unavailability. Third, concurrent medications and dental procedures were not matched in consideration when retaining the patient sample. Nevertheless, these variables have been adjusted for in the multivariable Cox regression models, and the results showed that the association between dental scaling and ESRD risk was virtually unaffected by these covariates. Fourth, our findings may not be generalizable to non-Asian populations [49]. The matched population, particularly those without scaling, may not align with those observed in clinical practice, due to the sample selection in the matching procedure. Fifth, our cohort was only followed up until the end of 2013, due to the regulations of the NHIRD. Nevertheless, prior studies have shown that over half of the events of ESRD occurred within 4 years after the diagnosis of CKD [50]. In our study, patients were followed up for a median of 8.1 years, and reliable estimated results could still be obtained in the context of survival analysis. Sixth, we did not consider the implementation of scaling after CKD diagnoses in the analyses. Previous studies have shown that the effects of periodontal therapy on systemic inflammation [22], endothelial function [23], and glomerular filtration rate [26,27] are not immediate. It requires 3 to 6 months for periodontal therapy or care to exert a protective effect on the periodontium and kidneys. Besides, as we have shown in the Results section, the duration between CKD diagnosis and ESRD was a median of 3.8 years for people receiving dental scaling treatment and 2.8 years for controls. If dental scaling within 2 years after CKD diagnosis is used to select and classify patients, a sizable proportion of subjects will be excluded because the ESRD happened prior to the receipt of dental scaling. Finally, because this study is observational, confounding by indication is possible. The severity of the periodontitis might form an indication for dental scaling, which potentially biased the distribution of severe periodontitis between two groups [51].

## 5. Conclusions

Dental scaling was associated with reduced risks of progression to ESRD, MACE, infections, and all-cause mortality in patients with CKD. Our findings suggest that dental scaling should be further promoted to improve the clinical outcome of CKD. Randomized controlled trials are warranted to examine the causal relationship of our findings.

## Figures and Tables

**Table 1:** Baseline characteristics of CKD cases with dental scaling treatment and matched controls.

Baseline Characteristic	Dental Scaling *n* = 33,637		Control *n* = 33,637		SDD
Age (years), mean (SD)	52.6	15.9	52.5	16.2	0.0062
Age group (years), *n* (%)					0.0145
20–34	5304	15.8	5490	16.3	
35–49	9563	28.4	9539	28.4	
50–64	10,396	30.9	10,383	30.9	
≥65	8374	24.9	8225	24.5	
Sex, *n* (%)					−0.0007
Male	16,941	50.4	16,951	50.4	
Female	16,691	49.6	16,681	49.6	
Insurance premium (USD/month), *n* (%)					−0.0184
0–500	15,829	47.1	15,658	46.6	
501–800	9517	28.3	9352	27.8	
≥801	8286	24.6	8622	25.6	
Stage 5 CKD, *n* (%)	5	0.01	7	0.02	−0.1855
Periodontal disease, *n* (%)	19,922	59.2	19,918	59.2	0.0003
Lifestyle factors, *n* (%)					
Smoking disorders	162	0.5	172	0.5	−0.0332
Alcohol abuse	456	1.4	472	1.4	−0.0193
Malnutrition	195	0.6	170	0.5	0.0761
Obesity	310	0.9	319	1.0	−0.0159
Comorbidity, *n* (%)					
Hypertension	12,945	38.5	13,022	38.7	−0.0053
Diabetes mellitus	8084	24.0	8148	24.2	−0.0057
Ischemic heart disease	5241	15.6	5316	15.8	−0.0093
Atherosclerosis	428	1.3	422	1.3	0.0079
Cardiac dysrhythmias	2474	7.4	2525	7.5	−0.0122
Heart failure	1196	3.6	1165	3.5	0.0150
Liver cirrhosis	481	1.4	489	1.5	−0.0092
Chronic obstruction pulmonary disease	3615	10.8	3528	10.5	0.0150
Cerebrovascular disease	2801	8.3	2813	8.4	−0.0026
Dyslipidemia	7531	22.4	7836	23.3	−0.0284
Malignancy	1704	5.1	1724	5.1	−0.0068
Mental disorders	8286	24.6	8463	25.2	−0.0155
Charlson Comorbidity Index					0.0096
0	4828	14.4	4959	14.7	
1	11,113	33.0	11,290	33.6	
2	6171	18.4	5955	17.7	
3	4508	13.4	4327	12.9	
≥4	7017	20.9	7106	21.1	
Systemic antibiotics, *n* (%)	13,264	39.4	12,167	36.2	0.0765
Statins, *n* (%)	3395	10.1	3926	11.7	−0.0899
Metformin, *n* (%)	4856	14.4	5151	15.3	−0.0382
Influenza vaccination, *n* (%)	262	0.8	850	2.5	−0.6587
Dental procedures, *n* (%)					
Subgingival curettage	770	2.3	983	2.9	−0.1382
Periodontal flap surgery	235	0.7	262	0.8	−0.0604
Other periodontal procedures	7022	20.9	11,702	34.8	−0.3882
Teeth extraction	10,770	32.0	7991	23.8	0.2278
Odontectomy	576	1.7	454	1.4	0.1333
Emergent dental care	6134	18.2	8564	25.5	−0.2350

CKD, chronic kidney disease; SD, standard deviation; SDD, standardized difference; USD, United States dollar.

**Table 2:** Risk of progression to ESRD and other complications for CKD cases receiving dental scaling treatment and matched controls.

	Dental Scaling		Control		Outcome Risk				
Clinical Outcome	Event, *n*	Crude Incidence Rate/1000 PY	Event, *n*	Crude Incidence Rate/1000 PY	IRR	cHR (95% CI)	*p*	aHR (95% CI) ^†^	*p*
Primary outcome									
Progression to ESRD	1296	4.61	1203	5.71	0.81	0.84 (0.78–0.91)	<0.0001	0.83 (0.77–0.90)	<0.0001
Secondary outcomes									
MACE ^‡^	5275	20.04	4493	22.68	0.88	0.92 (0.89–0.96)	<0.0001	0.91 (0.87–0.95)	<0.0001
Septicemia or sepsis	3592	13.05	3290	15.97	0.82	0.81 (0.77–0.85)	<0.0001	0.81 (0.77–0.85)	<0.0001
Urinary tract infection	4639	17.40	4125	20.67	0.84	0.88 (0.84–0.92)	<0.0001	0.87 (0.83–0.91)	<0.0001
Pyelonephritis	2017	7.33	1935	9.36	0.78	0.89 (0.83–0.95)	0.0002	0.87 (0.82–0.93)	<0.0001
Acute renal failure	1982	7.07	1978	9.41	0.75	0.75 (0.71–0.80)	<0.0001	0.76 (0.71–0.81)	<0.0001
All-cause mortality	1744	6.07	1576	7.29	0.83	0.79 (0.74–0.85)	<0.0001	0.81 (0.76–0.87)	<0.0001

aHR, adjusted hazard ratio; CI, confidence interval; cHR, crude hazard ratio; ESRD, end-stage renal disease; IRR, incidence rate ratio; MACE, major adverse cardiovascular events; PY, person-years. † Adjusted for age (linear), sex, insurance premium (categorical), coexisting diseases, lifestyle factors, current medications, and dental procedures listed in Table 1. ‡ Including acute myocardial infarction, new-onset heart failure, stroke, and cardiac dysrhythmias.

**Table 3:** Independent factors associated with ESRD.

Variable	aHR (95% CI)	*p*
Dental scaling	0.82 (0.75–0.88)	<0.0001
Insurance premium (USD/month)		<0.0001
501–800 vs. 0–500	0.84 (0.76–0.92)	0.0002
≥801 vs. ≥0–500	0.43 (0.37–0.49)	<0.0001
Stage 5 chronic kidney disease	106.72 (58.23–195.58)	<0.0001
Hypertension	2.00 (1.83–2.19)	<0.0001
Diabetes	2.39 (2.13–2.67)	<0.0001
Chronic obstruction pulmonary disease	0.75 (0.66–0.86)	<0.0001
Ischemic heart disease	0.83 (0.75–0.93)	0.0008
Cardiac dysrhythmias	0.69 (0.58–0.81)	<0.0001
Heart failure	1.61 (1.36–1.91)	<0.0001
Dyslipidemia	0.79 (0.71–0.87)	<0.0001
Obesity	0.44 (0.25–0.76)	0.0031
Mental disorders	0.70 (0.64–0.78)	<0.0001
Systemic antibiotics	0.79 (0.72–0.86)	<0.0001
Statins	1.66 (1.48–1.87)	<0.0001
Metformin	1.18 (1.05–1.32)	0.0062
Influenza vaccination	0.51 (0.32–0.82)	0.0058
Subgingival curettage	0.70 (0.51–0.96)	0.0282
Other periodontal procedures	0.71 (0.64–0.78)	<0.0001
Odontectomy	0.62 (0.35–1.09)	0.0963

aHR, adjusted hazard ratio; CI, confidence interval; USD, United States dollar.

**Table 4:** Subgroup analysis for the risk of ESRD, associated with dental scaling treatment.

Subgroup	DS or Control	Event, *n*	Crude Incidence Rate/1000 PY	IRR	cHR (95% CI)	*p*	aHR (95% CI) ^†^	*p*
Age <65 years	DS	834	3.87	0.75	0.79 (0.72–0.87)	<0.0001	0.79 (0.72–0.88)	<0.0001
	Control	829	5.14	reference	reference		reference	
Age ≥65 years	DS	462	7.02	0.92	0.95 (0.83–1.09)	0.4294	0.93 (0.81–1.07)	0.3187
	Control	374	7.60	reference	reference		reference	
Male	DS	642	4.61	0.80	0.84 (0.75–0.94)	0.0018	0.83 (0.74–0.93)	0.0010
	Control	604	5.76	reference	reference		reference	
Female	DS	654	4.61	0.81	0.84 (0.75–0.94)	0.0026	0.84 (0.75–0.95)	0.0037
	Control	599	5.67	reference	reference		reference	
Periodontal disease	DS	759	4.68	1.10	1.17 (1.04–1.32)	0.0099	1.13 (1.00–1.28)	0.0496
	Control	456	4.26	reference	reference		reference	
No periodontal disease	DS	537	4.52	0.63	0.64 (0.57–0.71)	<0.0001	0.64 (0.57–0.72)	<0.0001
	Control	747	7.22	reference	reference		reference	
Hypertension	DS	855	8.39	0.92	0.97 (0.87–1.07)	0.5206	0.96 (0.87–1.07)	0.4674
	Control	685	9.10	reference	reference		reference	
No hypertension	DS	441	2.46	0.64	0.66 (0.58–0.75)	<0.0001	0.68 (0.60–0.78)	<0.0001
	Control	518	3.83	reference	reference		reference	
Diabetes mellitus	DS	681	10.83	0.85	0.89 (0.79–0.99)	0.0347	0.89 (0.79–0.99)	0.0369
	Control	586	12.77	reference	reference		reference	
No diabetes mellitus	DS	615	2.82	0.75	0.77 (0.69–0.87)	<0.0001	0.80 (0.71–0.90)	0.0002
	Control	617	3.75	reference	reference		reference	
Systemic antibiotics	DS	485	4.34	0.98	1.02 (0.89–1.18)	0.7876	0.99 (0.86 –1.15)	0.9352
	Control	330	4.43	reference	reference		reference	
No systemic antibiotics	DS	811	4.78	0.75	0.78 (0.71–0.86)	<0.0001	0.78 (0.70 –0.86)	<0.0001
	Control	873	6.41	reference	reference		reference	
Statins	DS	280	11.19	0.83	0.86 (0.73–1.02)	0.0876	0.85 (0.71–1.02)	0.0725
	Control	268	13.54	reference	reference		reference	
No statins	DS	1016	3.97	0.81	0.84 (0.77–0.92)	0.0001	0.83 (0.76–0.91)	<0.0001
	Control	935	4.90	reference	reference		reference	
Metformin	DS	440	11.96	0.84	0.86 (0.75–0.99)	0.0292	0.86 (0.74–0.99)	0.0308
	Control	397	14.27	reference	reference		reference	
No metformin	DS	856	3.50	0.79	0.83 (0.75–0.91)	0.0001	0.83 (0.75–0.92)	0.0003
	Control	806	4.41	reference	reference		reference	

aHR, adjusted hazard ratio; CI, confidence interval; cHR, crude hazard ratio; DS, dental scaling; IRR, incidence rate ratio. † Adjusted for age (linear), sex, insurance premium (categorical), coexisting diseases, lifestyle factors, current medications, and dental procedures listed in Table 1.

## Data Availability

The data presented in this study are available on request from the corresponding author.

## Supplementary Materials

The following are available online at https://www.mdpi.com/article/10.3390/ijerph18178910/s1, Table S1: ICD-9-CM codes of covariates and outcomes, Table S2: Risk of ESRD in patients with dental scaling who had various dental treatments, Table S3: Interaction analysis for risk of ESRD associated with dental scaling.
