# Supplementary material for: Association between Dental Scaling and Reduced Risk of End-Stage Renal Disease: A Nationwide Matched Cohort Study
Source: Int J Environ Res Public Health. 2021 Aug 24;18(17):8910. doi: 10.3390/ijerph18178910 (PMC8430582; doi:10.3390/ijerph18178910)
Supplement: Supplementary file 1 [file ijerph-18-08910-s001.zip › ijerph-1306499-supplementary.pdf]

Table S1. ICD-9-CM codes of covariates and outcomes.

**Coexisting disease**	
Chronic kidney disease	250.4, 271.4, 274.1, 403-404, 572.4, 580-589, 590-591
Periodontal disease	523.0, 523.1, 523.3, 523.4
Hypertension	401-405
Diabetes mellitus	250
Ischemic heart disease	410-414
Atherosclerosis	440
Cardiac dysrhythmias	427
Heart failure	428
Liver cirrhosis	571.2, 571.5, 571.6
Chronic obstructive pulmonary disease	490, 491, 496
Cerebrovascular disease	430-438
Dyslipidemia	272.0-272.4
Malignancies	140-208, 230-234
Mental disorders	290-319
**Lifestyle factor**	
Smoking	V15.82, 305.1
Alcohol abuse	291.0, 291.1, 291.2, 291.3, 291.4, 291.5, 291.8, 291.81, 291.82, 291.89, 291.9, 303.00-303.03, 303.90-303.93, 305.00-305.03, 357.5, 425.5, 535.30, 535.31, 571.0, 571.1, 571.2, 571.3, E860.0
Malnutrition	262, 263.0, 263.1, 263.8, 263.9, 783.22, 783.21, 799.4, V85.0, 260, 261
Obesity	278
**Outcome**	
Acute myocardial infarction	410
Heart failure	428
Stroke	430-437
Cardiac dysrhythmias	427
Septicemia or sepsis	038, 790.7, 995.92
Urinary tract infection	599.0
Pyelonephritis	590
Acute renal failure	584

Table S2. Risk of ESRD in patients with dental scaling who had various dental treatments.

**Dental Treatment**	**Event, *n***	**Crude Incidence Rate/1,000 PY**	**IRR**	**cHR (95% CI)**	***p***	**aHR (95% CI)†**	***p***
No dental scaling	1203	5.71	1.00 (reference)	1.00 (reference)		1.00 (reference)	
Patients with dental scaling who had							
Subgingival curettage	26	4.25	0.74	0.77 (0.52 – 1.14)	0.1897	0.80 (0.54 – 1.18)	0.2575
Periodontal flap surgery	8	4.26	0.75	0.78 (0.39 – 1.55)	0.4732	0.91 (0.45 – 1.82)	0.7803
Teeth extraction	477	5.53	0.97	1.00 (0.90 – 1.11)	0.9589	0.92 (0.82 – 1.02)	0.1226
Odontectomy	9	1.90	0.33	0.35 (0.18 – 0.67)	0.0016	0.67 (0.35 – 1.30)	0.2353
Emergency dental care	199	4.12	0.72	0.75 (0.64 – 0.87)	0.0001	0.86 (0.74 – 1.01)	0.0680

aHR, adjusted hazard ratio; CI, confidence interval; PYs, person-years. † Adjusted for age, sex, monthly premium, coexisting diseases, lifestyle factors, and current medications.

Table S3. Interaction analysis for risk of ESRD associated with dental scaling.

**Variables**	***p*†**
**Age**	<0.0001
**Sex**	0.9561
**Periodontal disease**	0.6633
**Hypertension**	<0.0001
**Diabetes mellitus**	<0.0001
**Systemic antibiotics**	0.0901
**Statins**	<0.0001
**Metformin**	<0.0001

† *p* for hazard ratio interaction.
